# The impact of foreign player types on lineup efficiency in the Chinese basketball association

**DOI:** 10.3389/fspor.2026.1851077

**Published:** 2026-10-14

**Authors:** Zengyu Li, Carlos Lago Peñas, Mingjia Qiu, Feiyu Ren, Mingxin Zhang

**Affiliations:** 1 School of Athletic Performance, Shanghai University of Sports, Shanghai, China; 2 Shanghai Key Lab of Human Performance, Shanghai University of Sport, Shanghai, China; 3 Faculty of Education and Sport Sciences, Universidade de Vigo, Pontevedra, Spain

**Keywords:** basketball, foreign players, lineup, performance analysis, player role, random forest

## Abstract

The inclusion of foreign players has raised the competitive level of basketball leagues and broadened the skill sets of local athletes. However, the effects of lineups combining local and foreign players on offensive and defensive performance remain understudied. This study examined role definitions, interactions, and compatibility patterns between local and foreign players in the CBA. Data from 34,963 lineups across three seasons (2021–2024) involving 739 players were analyzed. Through Gaussian mixture model and random forest model analysis, 10 local player types and 7 foreign player types were identified. Association rule analysis of regular local lineups revealed: (1) a ball-handler and defensive–offensive paint anchor frequently co-occur with a “3D player”; (2) coaches favor functionally diverse local lineups over multiple ball-handlers; and (3) players with overly narrow skill profiles are rarely included in regular lineups. The decision tree model revealed that local-player combinations paired with a “heavy big” increased effective field goal percentage; pairing with a dominant shooting guard reduced offensive turnover percentage; pairing with a versatile big improved offensive rebound percentage and both offensive and defensive free throw rates; and pairing with a mid-to-long-range scoring swingman lowered opponents' offensive rebound percentage, reduced their effective field goal percentage, and increased their turnover percentage.

## 1 Introduction

Basketball is one of the most widely followed sports worldwide, and match outcomes depend not only on individual skills but also on lineup construction and player synergy (1). As the modern game increasingly values versatility, researchers have developed functional role classifications based on tracking data, since players often perform multiple tasks depending on game context (2, 3). Several studies have applied clustering methods to define player roles, including the probabilistic role assignment proposed by Kalman and Bosch (4) and the classification of foreign and local players by Wang (5). However, classifications based primarily on conventional performance statistics may not fully capture the functional diversity of player roles (6). More recent work has extended role classification by incorporating both offensive and defensive play-type information (7). As player functions become increasingly differentiated, spatial indicators such as shooting distance may further distinguish players with similar play-type profiles by capturing differences in shot selection. More differentiated role classifications may, in turn, provide a stronger basis for examining functional complementarity within lineups.

Lineup research has emphasized tactical compatibility and complementary skill sets (1, 8). From a functional-role perspective, lineup performance may therefore depend not only on the characteristics of individual role types but also on the compatibility among the roles represented within a lineup. Methods such as classification trees and network models have helped identify performance patterns, including the effectiveness of ball screens (9) and key predictors of winning (10), and anthropometric and technical indicators have also been examined (5). Random-forest models have also been used to characterize complex relationships among performance variables (11). Such multivariate approaches are particularly relevant when lineup outcomes may reflect interactions among multiple player roles rather than isolated player characteristics.

The integration of foreign players has become common across global sports (12) and has been associated with variation in offensive and defensive performance in basketball leagues (13). In the Chinese Basketball Association (CBA) league, the top 16 teams are subject to a “7-player-period” rule, which restricts foreign players to a maximum of seven player appearances across four quarters, whereas the four lowest-ranked teams from the previous season are allowed an “8-player-period” quota. This regulation is designed to maintain competitive balance while promoting opportunities for the development of domestic players. Within this regulatory context, foreign-player recruitment and deployment constitute an important component of team strategy (14), most studies have focused on social issues such as discrimination, labor migration, and management (13, 15), leaving limited evidence on how combinations of local and foreign players contribute to lineup effectiveness under current regulations. Accordingly, the relationship between foreign-player participation and lineup performance may depend on the functional compatibility between foreign-player roles and the domestic-role structure, rather than on foreign-player ability alone.

To address this gap, this study examines association rules among local player roles and evaluates whether offensive and defensive lineup outcomes differ when recurrent local-role combinations are paired with different foreign-player types within the current CBA policy framework. We hypothesize that certain combinations of local roles form recurrent patterns and that lineup efficiency differs across pairings between these local-role combinations and specific foreign-player types. By integrating player-role classification with lineup-level analysis, this study extends role-based performance analysis from the identification of individual player types to the examination of functional compatibility between local and foreign-player roles. The findings may assist coaches and performance staff in lineup design and tactical preparation and may also provide empirical context for discussions of foreign-player regulation and tactical diversity in professional basketball.

## 2 Sample and methods

### 2.1 Data collection

The dataset included all regular-season games from the 2021–2024 CBA seasons. Player height and weight were obtained from the official CBA website (https://www.cbaleague.com/data/), and body mass index (BMI) was calculated as weight (kg)/height (m)². Play-type data covered all scoring categories, enabling the quantification of team tactical behavior (1). Defensive play types were classified according to the corresponding defensive categories of each offensive play type. The calculation procedure followed the same approach as that used for offensive play types. The defensive play-type data were obtained from Synergy Sports Technology (2), and the reliability and validity of this classification system have been previously demonstrated, with high inter-observer agreement and strong consistency with actual match tactics reported by Chen (3). Offensive play-type data were provided by Root Sports, the CBA's official data intelligence partner, which uses AI developed by the Institute of Automation at the Chinese Academy of Sciences to generate advanced performance data. However, due to the unavailability of data for Cut, Put Backs, and Transition in the defensive categories on the Synergy website, we ultimately utilized 10 offensive and 7 defensive play types as our final clustering variables, as shown in Table 1. To characterize player-specific performance profiles across different offensive and defensive play types and to distinguish players with similar tactical roles based on play-type usage and efficiency, we extracted the usage percentage (Usage%) and points per possession for each play type (PPPP). PPPP was calculated in Equation 1:

$$\mathrm{PPPP}=\frac{\mathrm{Total}\;\mathrm{field}\;\mathrm{goals},\;\mathrm{turnovers},\;\mathrm{and}\;\mathrm{fouls}\;\mathrm{drawn}\;\mathrm{in}\;\mathrm{this}\;\mathrm{play}\;\mathrm{type}}{\mathrm{Overall}\;\mathrm{field}\;\mathrm{goals},\;\mathrm{turnovers},\;\mathrm{and}\;\mathrm{fouls}\;\mathrm{drawn}}$$ (1)

**Table 1:** Operational definitions of the variables used in the analysis.

Variable	Definition
Height	The vertical distance from the top of the head to the ground when a person is standing upright.
Weight	The total weight of all parts of the human body (including bones, skeletal muscles, joints, ligaments, adipose tissues, etc.)
Body mass index	BMI = weight (kg)/height (m)²
Spot Up	The end of the possession is marked by a successful catch-and-shoot shot or a successful catch-and-drive play.
Handoffs	The screener holds the ball, passes it to an approaching teammate ready to receive the ball, and provides a timely screen for him.
Isolation	The end of the possession is marked by the player who has the ball successfully completed a one-on-one match.
Off Screen	The player without the ball uses his teammates' cover to move from the position near the basket to the sideline.
Post Up	The player holds the ball and initiates an attack or sets up for an assist at a position less than 15 inches away from the basket.
P&R Ball Handler	The screen is set up beside the player with the ball. Then, the player uses the screen to launch an attack.
P&R Roll Man	After completing the screen for the ball handler, the roll man moves towards the basket by either cutting down or bouncing the ball and catching it to finish the play.
Transition	The end of a possession is marked by the defending team gaining possession of the ball and switching from defense to offense.
Cut	The players take advantage of the time difference to complete the shots at positions close to or slightly away from the basket.
Put Backs	After grabbing the rebound, this refers to the attempt to score before choosing to pass the ball or go to the basket for an offensive move.
Rim	The distance within 5 feet of the basket
Mid-Range	Field goal attempts taken outside the paint but inside the three-point line.
Three-Point	The standard distance from the basket as stipulated by the International Basketball Federation (FIBA) is 6.75 meters.
Offensive rating (ORtg)	Points scored per 100 possessions for team.
Defensive rating (DRtg)	Points allowed per 100 possessions for team.
Net rating (NetRtg)	The difference between the total points scored and the total points lost over 100 rounds.
Effective Field Goal Percentage (eFG%)	The shooting percentage calculated by converting three-point shots into two-point shots，it directly reflects the level of a team or player's efficiency in offensive shooting during the game.
Turnover Percentage (TOV%)	The proportion of possessions ended by turnovers to the total number of offensive possessions of the team.
Offensive Rebound Percentage (ORB%)	The offensive rebounding rate of a team refers to the proportion of offensive rebounds a team secures out of all offensive rebounding opportunities.
Free Throw Rate (FTr%)	Dividing a team's free throws made by field goal attempts represents simultaneously the team's ability to get to the foul line and ability to make foul shots.

### 2.2 Study participants

During the 2021–2024 Chinese Basketball Association (CBA) seasons, 1,813 players were registered. Repeated appearances of the same player across different seasons were treated as separate samples, as player characteristics and performance profiles may vary between seasons. Players who appeared in fewer than 10 games or averaged under 5 min per game were considered unrepresentative (16) and excluded, resulting in a final sample of 739 players. Across the three seasons, 154 players participated in one season, 87 players participated in two seasons, and 110 players participated in all three seasons. To examine player-role combinations, data from 34,963 lineup configurations across the three seasons were collected. Following the definition in Basketball Analytics by Shea, lineups with over 100 min of cumulative regular-season playing time are classified as “regular lineups,” and the players in these lineups are considered part of the main rotation (6).

### 2.3 Reliability assessment of play-type data

To assess the reliability of the play-type data obtained from the database, 10 games from the 2021–2024 seasons were selected for independent video analysis. Each game was independently reviewed by two basketball video analysts, each with 5 years of professional experience. Following the play-type definitions provided by the database, the analysts reviewed and quantified offensive and defensive play-type events and subsequently calculated the corresponding play-type Usage% and points per possession for each play type (PPPP) using the predefined formulas. The video-derived Usage% and PPPP values were then compared with the corresponding database-derived values from the same games. Agreement between the two analysts, as well as agreement between the video-derived and database-derived measures, was assessed using two-way mixed-effects intraclass correlation coefficients (ICCs) with absolute agreement, with 95% confidence intervals (CIs) calculated for each estimate. Detailed reliability results are presented in Table 2.

**Table 2:** Reliability of offensive and defensive play-type measures derived from video analysis and the database.

Play-type category	possessions	Inter-analyst Usage% ICC (95% CI)	Inter-analyst PPPP ICC (95% CI)	Video-derived vs. database Usage% ICC (95% CI)	Video-derived vs. database PPPP ICC (95% CI)
Offensive	917	0.95 (0.92–0.97)	0.93 (0.89–0.96)	0.93 (0.89–0.96)	0.91 (0.86–0.95)
Defensive	894	0.93 (0.91–0.95)	0.92 (0.90–0.94)	0.92 (0.90–0.93)	0.90 (0.88–0.93)

### 2.4 Statistical analysis

A Gaussian mixture model (GMM) was used to cluster players and classify their roles. Clustering features included anthropometric indicators, shooting-distance metrics, and offensive and defensive play types, enabling differentiation among players with similar styles. Anthropometric variables were included to characterize body-size attributes relevant to player function, shooting-distance metrics to capture spatial scoring tendencies, and offensive and defensive play-type measures to represent tactical involvement and efficiency. Prior to clustering, all continuous variables were standardized to z-scores to minimize the influence of differences in measurement scale. The GMM estimates the parameters of each Gaussian distribution and computes the probability of each data point belonging to them (7), with parameter estimation performed via maximum likelihood. The probability density function of the GMM is shown in Equation 2. The optimal clustering solution was determined by silhouette coefficient values, with values closer to 1 indicating stronger cohesion and separation, and negative values reflecting poor clustering quality (17). To further evaluate the discriminative validity of the player classifications derived from the Gaussian mixture models, random forest classifiers were developed separately for domestic and foreign players. The cluster membership was specified as the outcome variable, whereas the original performance indicators used in the clustering procedure were entered as predictors. The data were randomly divided into a training set (70%) and a testing set (30%). Classification performance was evaluated using the overall accuracy in the held-out testing set. Variable importance was quantified using the mean decrease in accuracy and mean decrease in Gini impurity, with higher values indicating a greater contribution to distinguishing among player types.

$$\begin{array}{c} p(x)=\sum_{i\;=\;1}^{K}\;\phi_{i}N(x|\mu_{i},\Sigma_{i}),\sum_{i\;=\;1}^{K}\;\phi_{i}=1,\phi_{i}\;\geq 0 \end{array}$$ (2)

The frequent pattern growth (FP-growth) algorithm was used to identify associations among local player-role combinations (9). Association-rule analysis was conducted at the role level rather than at the level of individual player identity. After clustering, each local player was represented by the assigned role category, and FP-growth was used to identify recurrent combinations of local player roles within single-foreign-player lineups. Because these lineups comprised one foreign player and four local players, four-role local combinations were evaluated first. However, none of the resulting four-role association rules satisfied the prespecified confidence, and lift criteria. The analysis therefore focused on three-role combinations, which represented the highest-order local-role configurations that met the association-rule thresholds while retaining a recurrent local-role core within the five-player lineup. FP-growth constructs a prefix tree (FP-tree) from the dataset and requires only two scans, reducing memory usage. Associations were evaluated using support, confidence, and lift. Support represents the proportion of records containing both A and B relative to the entire dataset:

Support(A→B)=(A∩B)/N

where N is the total number of lineup records, and A ∩ B is the number of records in which antecedent A and consequent B co-occur.

Confidence represents the conditional probability that consequent B occurs in records containing antecedent A and is expressed as follows:

Confidence(A→B)=Support(A→B)/Support(A)

Lift compares the observed co-occurrence of A and B with the co-occurrence expected if A and B were statistically independent and is expressed as follows:

Lift(A→B)=Support(A→B)/[Support(A)×Support(B)]

Lift values > 1 indicate that A and B co-occur more frequently than expected under independence. Multi-item results were used to identify recurrent patterns in player-role configurations. Association rules were applied to three-role local combinations, using a minimum lift > 1 and confidence > 65% to retain positive associations (10).

A decision tree based on the classification and regression tree (CART) algorithm was then used to examine foreign player combinations. Cost-complexity pruning was applied, with the optimal pruning parameter (ccp_alpha) selected using five-fold cross-validation. Cross-validation performance was evaluated using the root mean squared error (RMSE). Foreign player presence (on-court/off-court) was one-hot encoded as the independent variable, while offensive rating, defensive rating, net rating, and the four factors were used as dependent variables to evaluate lineup performance across different offensive and defensive scenarios.

## 3 Results

### 3.1 Clustering results and redefined player types

The clustering solution for domestic players yielded a silhouette coefficient of 0.32, indicating an acceptable clustering structure characterized by moderate within-cluster cohesion and between-cluster separation. In comparison, the clustering solution for foreign players achieved a higher silhouette coefficient of 0.44, suggesting improved cluster validity, with greater internal homogeneity and clearer separation among clusters. The random forest classifier achieved a testing accuracy of 79.07% for domestic players and 83.33% for foreign players as shown in Figure 1. These findings indicate that the player types identified by the Gaussian mixture models could be distinguished with relatively high accuracy using the original performance indicators, with particularly strong discriminative performance observed for foreign-player classifications. The variable-importance analysis further identified the indicators that contributed most strongly to differentiating among player types, as reflected by higher mean decreases in accuracy and Gini impurity. Local players (marked as C) are as shown in Figure 2 were classified into ten roles based on the clustering results, while foreign players (marked as F) were classified into seven roles as shown in Figure 3. C1 refers to a ball-handler who primarily relies on isolation plays, pick-and-roll ball-handling, drives to the basket, and mid-range shooting. C2 represents a transition-oriented swingman who is particularly effective in transition offense, spot-up shooting, and three-point shooting out of handoffs. C3 denotes a guard shooter with offensive strengths in spot-up shooting and three-point shooting in transition. C4 refers to an offensive and defensive anchor in the paint who specializes in screen setting, low-post offense, and mid-range shooting. These players are also effective at defending pick-and-roll actions. C5 represents a 3–4 swingman with offensive versatility in pick-and-pop and spot-up situations. Defensively, this player type is effective against spot-up, off-screen, post-up, and pick-and-roll actions. C6 represents a stretch big characterized by relatively small size and strong mid- and long-range shooting, particularly in pick-and-pop situations. C7 refers to a three-point-oriented shooting guard who excels at three-point shooting, particularly from catch-and-shoot opportunities and off-screen actions, and relies heavily on off-ball movement. C8 denotes a 3-and-D player, referred to as a “3D player” throughout this study, who specializes in three-point shooting and is particularly effective at defending isolation plays. C9 represents a traditional big man, typically the tallest center, with strengths in low-post offense, pick-and-roll actions, and defending opponents' low-post play. C10 refers to a defensive guard whose primary role is to defend perimeter players.

**Figure 1:**
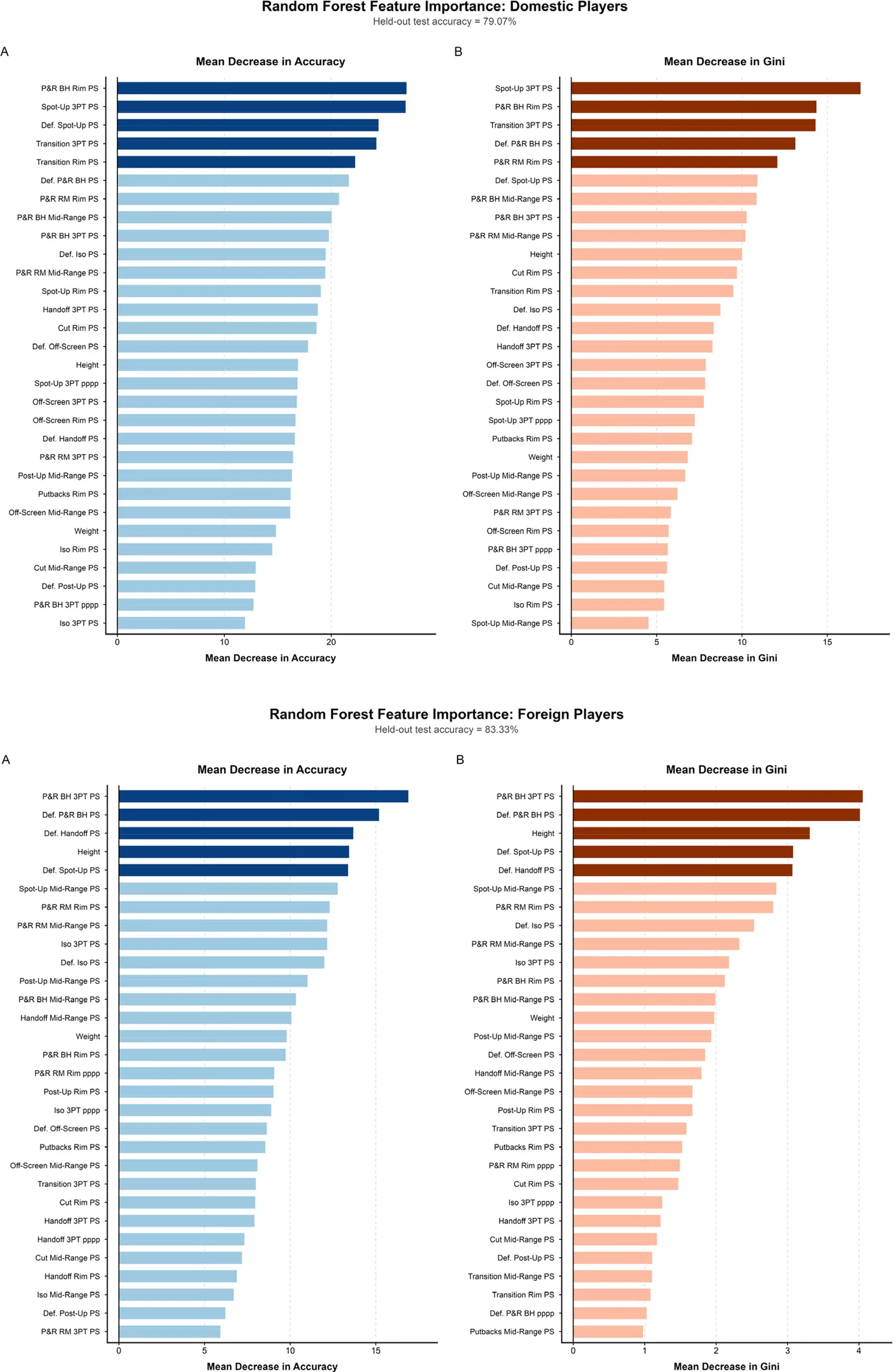
Random forest feature importance for classifying foreign and domestic player types.

**Figure 2:**
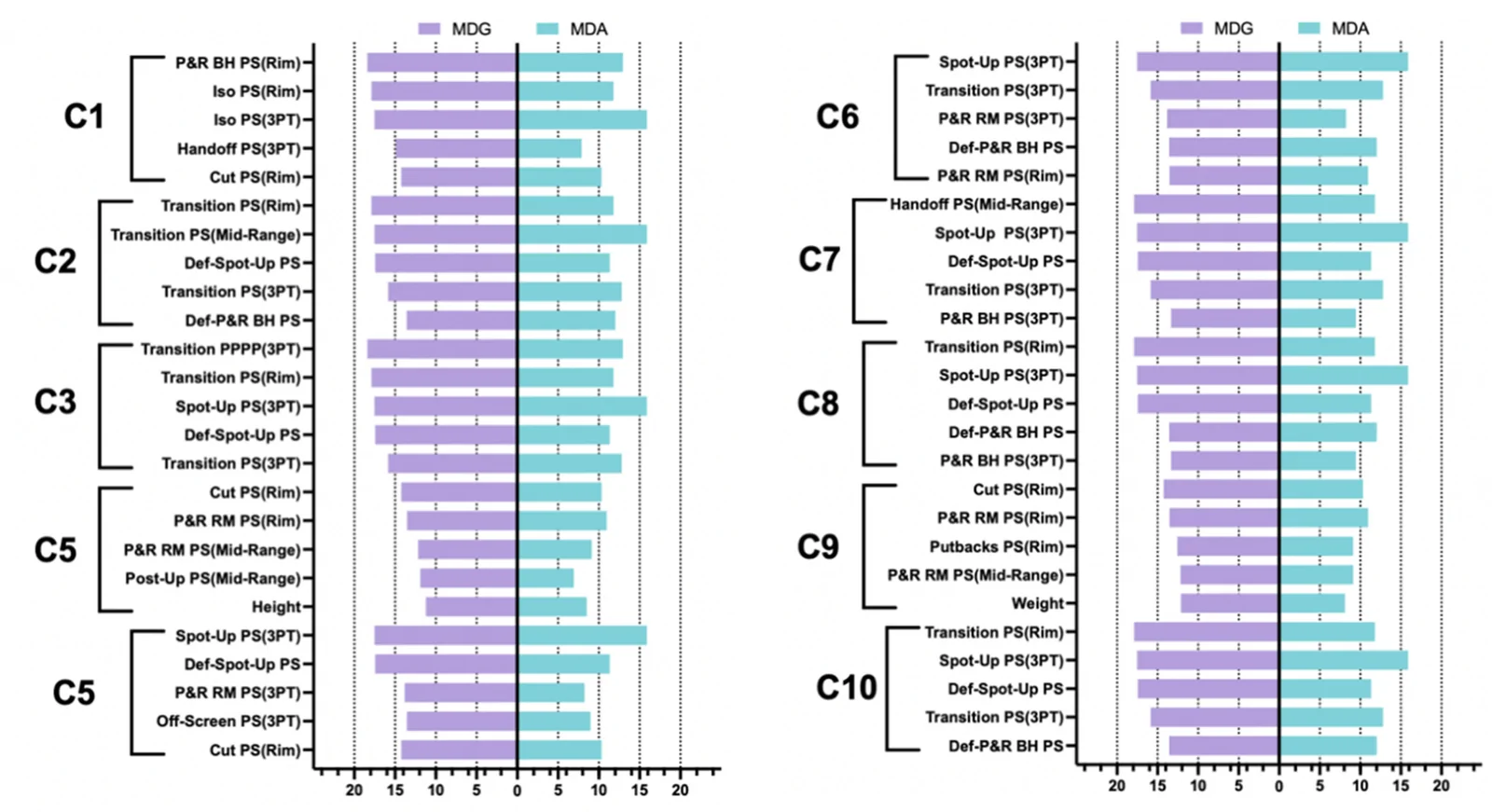
Random forest variable-importance plot showing the five most important indicators for distinguishing domestic-player roles.

**Figure 3:**
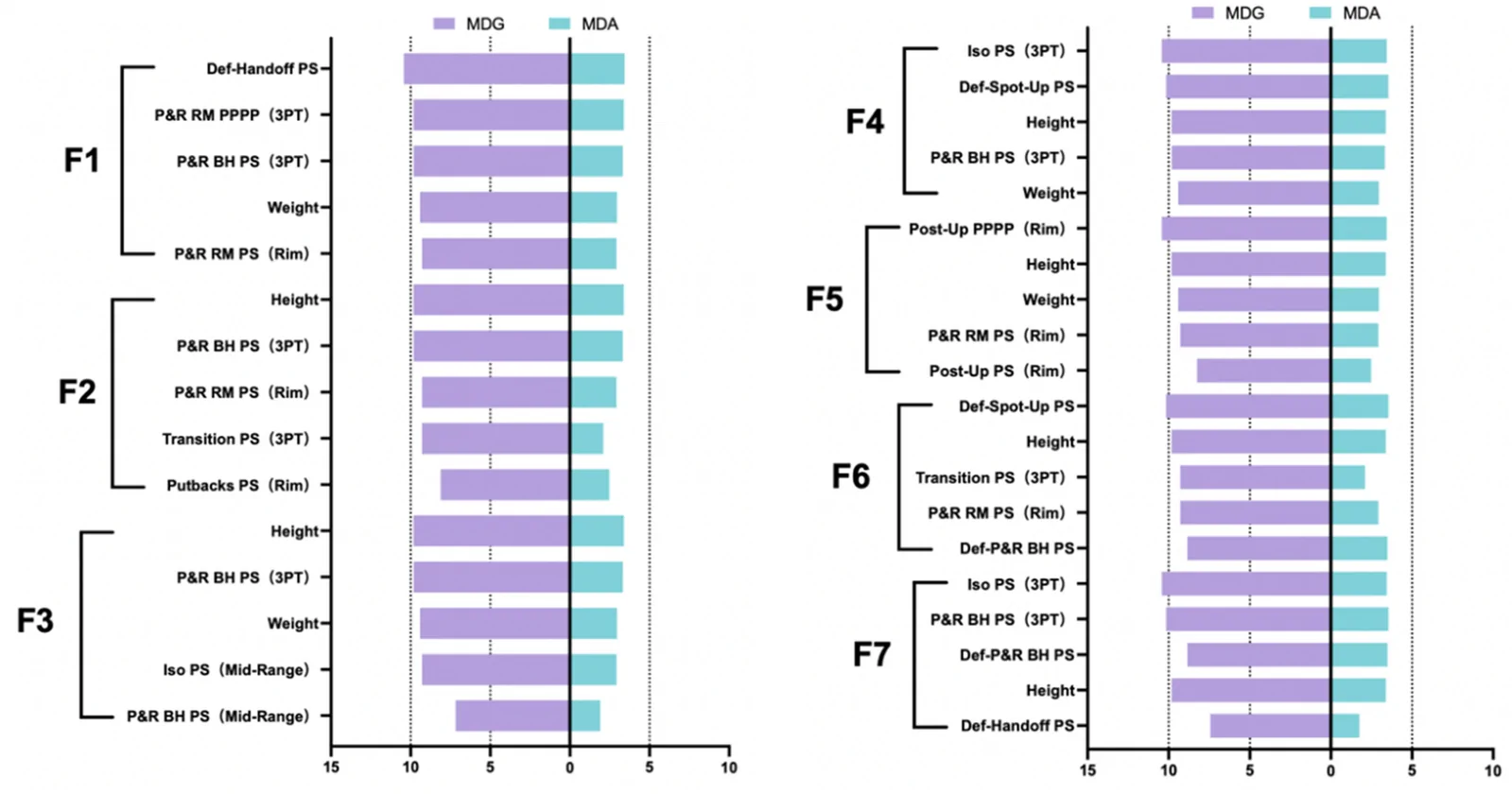
Random forest variable-importance plot showing the five most important indicators for distinguishing foreign-player roles.

F1 represents a versatile big characterized offensively by low-post scoring, pick-and-roll finishing, and long-range shooting, and defensively by the ability to guard both pick-and-roll actions and low-post offense. F2 represents a scoring forward who primarily attacks the basket through face-up drives, while also demonstrating defensive versatility against wings and hybrid forwards. F3 represents a mid- to long-range scoring swingman characterized by pull-up and spot-up shooting, transition offense, and perimeter defense. F4 represents a rim-running big characterized by good mobility, athletic interior finishing, and pick-and-roll finishing, with defensive effectiveness against opponents' pick-and-roll actions. F5 represents a heavy big characterized by a strength-based interior playing style, with prominent roles in low-post offense and screen setting. F6 represents a facilitating guard, characterized by a ball-dominant playmaking role, pick-and-roll creation, mid-range scoring, and active point-of-attack defense. Finally, F7 represents a dominant scoring guard, characterized by high offensive involvement, perimeter shot creation, three-point shooting, downhill attacks, and relatively efficient ball control.

### 3.2 Association rules among domestic player roles

Because player clusters were incorporated into lineup combinations, multiple observed five-player lineups could correspond to the same role-based lineup structure. Therefore, lineup performance statistics were aggregated and standardized to a per-48-minute basis to account for differences in playing time exposure and to characterize the performance profiles of different lineup combination types.

Based on Table 3, several stable local player combinations emerged. When C10 (point guard) and C4 (offensive and defensive anchor in the paint) played together, they were typically paired with C8 (3D player), the rule had the greatest support (0.2215), identifying C1–C4–C8 as the most recurrent three-role structure. In contrast, when C2 (transition-oriented swingman) and C8 (3D player) were on the court, the lineup commonly included C4, and this combination has the highest confidence (0.8333). When C5 (3–4 swingman) and C6 (stretch big) appeared together, they were usually paired with C1 (ball-handler) and yield highest lift (1.1789).

**Table 3:** Association rules among domestic player roles.

Rules	Degree of support	Degree of confidence	Coverage	Degree of lift	Count
{C10, C4} => {C8}	0.0503	0.8333	0.0604	1.1659	15
{C2, C8} => {C4}	0.0570	0.6538	0.0872	1.1134	17
{C5, C6} => {C1}	0.0604	0.6923	0.0872	1.1789	18
{C1, C4} => {C8}	0.2215	0.7333	0.3020	1.0260	46

### 3.3 Pairing rules and configurations

Following initial screening based on playing time criteria and the consolidation of identical lineup combinations according to player clustering results, 299 regular lineup combinations were identified. Because C1–C4–C8 had the greatest support and count among the qualifying domestic-role rules (Table 3), the CART analysis focused on lineup combinations in which these three domestic roles were simultaneously present, yielding 46 lineup combinations for further analysis. The final CART models were restricted to a maximum depth of 3, with a minimum of 2 observations required for node splitting and at least 4 observations retained in each terminal leaf. Five-fold cross-validation identified an optimal cost-complexity pruning parameter (ccp_alpha) of 0.004, with cross-validated RMSE values ranging from 0.065 to 0.072. These results indicate that the CART models should be interpreted as exploratory representations of role-based lineup patterns given the limited analytical sample (*n* = 46), rather than as evidence of stable predictive performance or causal relationships. As shown in Figure 4, the trio of C1 (ball-handler), C8 (3D player), and C4 (offensive and defensive anchor in the paint) achieved the highest offensive efficiency when paired with a heavy interior player. Mid-to-long-range foreign scorers were the most effective for boosting defensive efficiency is presented in Figure 5. Pairing with a heavy big produced the highest offensive EFG%, while pairing with a dominant shooting guard lowered the team's turnover rate. A versatile big increased DRB% and free-throw efficiency on both ends. Pairing with a mid- to long-range scoring swingman raised ORB%, reduced opponents' EFG%, and increased their turnover rate.

**Figure 4:**
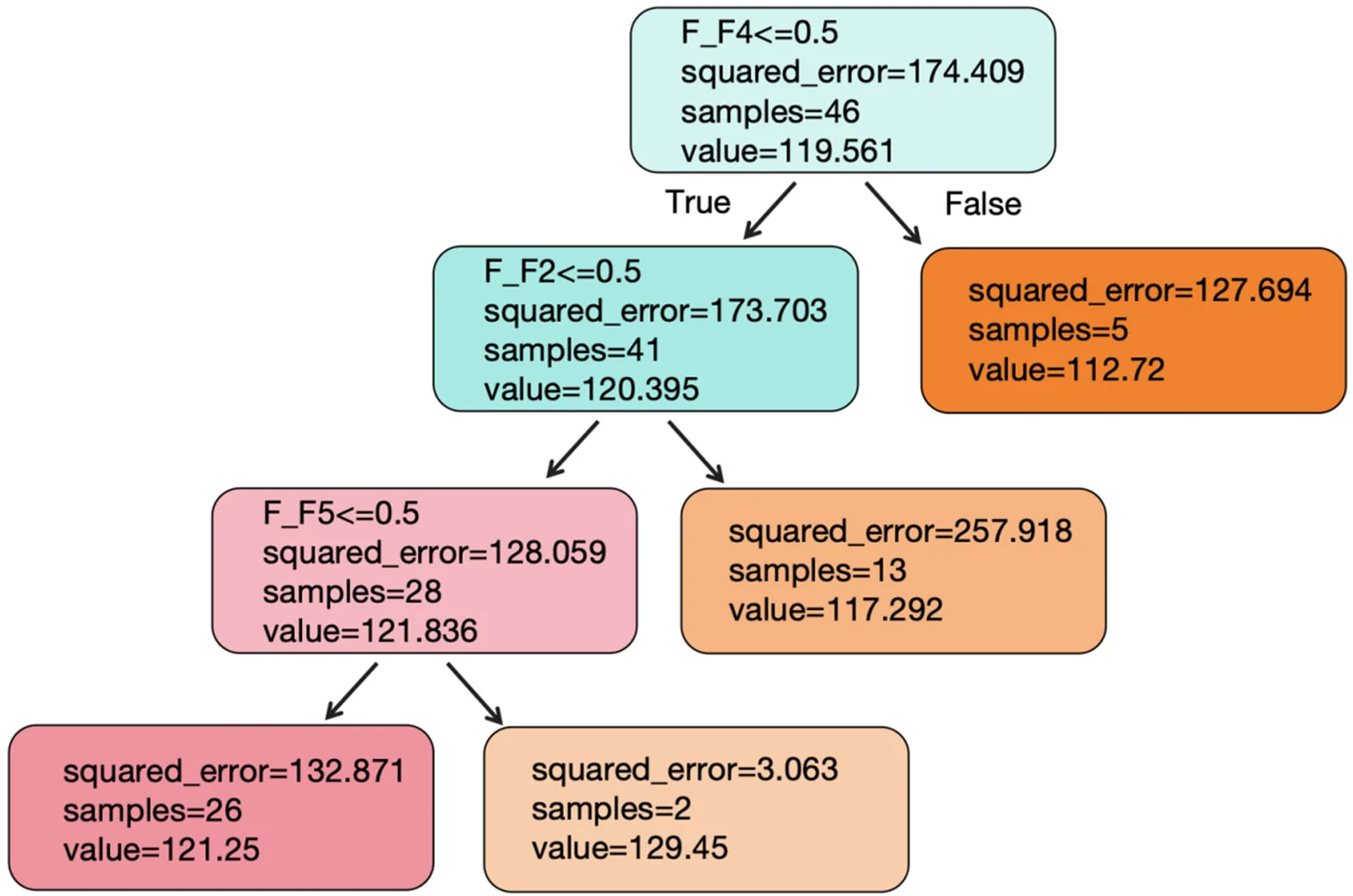
Decision tree model showing offensive efficiency in the CBA generated by classification and regression tree (CART) analysis.

**Figure 5:**
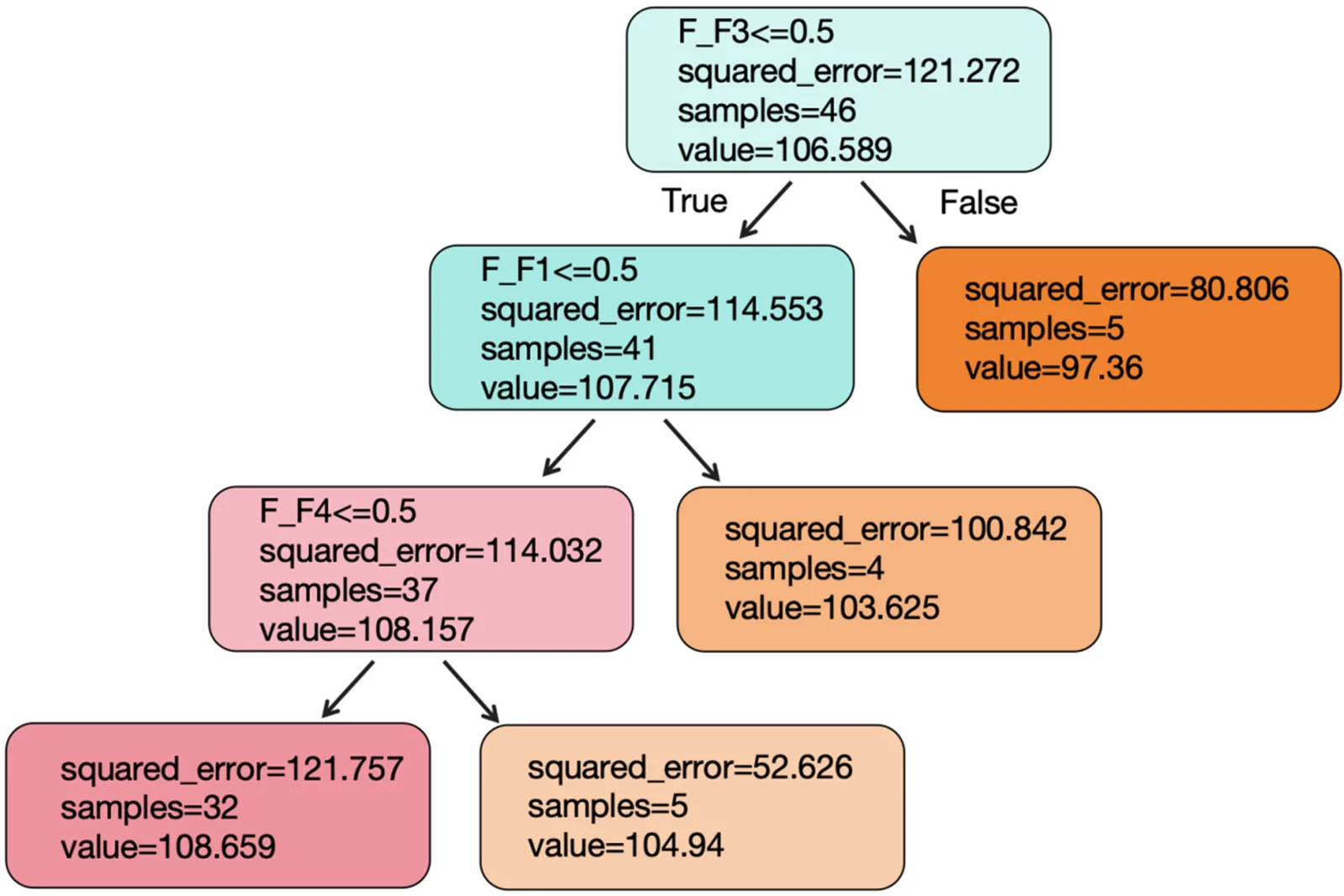
Decision tree model showing defensive efficiency in the CBA generated by classification and regression tree (CART) analysis.

## 4 Discussion

This study identified 10 data-driven local-player profiles and seven foreign-player profiles using anthropometric, shooting-distance, and play-type variables. Association-rule analysis identified recurrent local-role configurations, whereas CART analysis characterized how lineup-efficiency indicators varied when these configurations were paired with different foreign-player profiles. Given the observational design and the exploratory nature of the CART models, the findings are interpreted as conditional associations within the analyzed sample rather than causal effects.

### 4.1 Distinct classification patterns of local and foreign players

The distinct clustering patterns of local and foreign players may reflect differences in functional role allocation within the CBA rather than nationality *per se*. Under the foreign-player participation restrictions applied during the study period, imported players were often recruited to fulfill specific high-leverage functions or address roster and matchup needs (5). Accordingly, anthropometric characteristics and defensive play types may have contributed more strongly to differentiating foreign-player profiles because size, mobility, and defensive versatility influence rim protection, switching, and perimeter coverage (11). By contrast, local players formed the more stable lineup core and were distributed across a broader range of offensive responsibilities, which may explain why execution efficiency across play types contributed more strongly to their classification. These differences should not be interpreted as intrinsic nationality effects, because team strategy, player experience, injury status, and role assignment may also shape the observed profiles and the relative contributions of local and foreign players (12).

### 4.2 Local player association rules

Association-rule analysis identified recurrent local-role combinations centered on interior presence and complementary perimeter functions. The prominence of paint anchors is consistent with evidence that local centers can contribute to both offensive organization and defensive stability (5, 18), whereas recurrent pairings involving stretch forwards may reflect the spacing and transition demands of contemporary CBA play (13). Functionally, this configuration integrates on-ball creation, screening and interior play, floor spacing, and perimeter defense. Such a configuration may reduce functional redundancy: the ball handler assumes on-ball creation, the paint anchor provides screening, rebounding, and interior defense, and the 3D player contributes off-ball spacing and defensive versatility (15). The recurrent co-occurrence of these roles therefore appears more consistent with functional complementarity than with the independent superiority of any single player type.

### 4.3 Associations between foreign-player roles and lineup-efficiency indicators

The CART analysis suggested that lineup-efficiency profiles differed according to the foreign-player role paired with the recurrent local-player core. Because these differences were node-specific and derived from a limited analytical sample, they are best interpreted as conditional patterns that may reflect how particular foreign-player functions complement specific requirements of the local-role structure.

Lineups paired with heavy bigs showed higher offensive eFG%. One possible explanation is that the low-post and screening roles typically associated with this player profile may alter the spatial organization of the offense and the distribution of shot attempts. Establishing position close to the basket and participating in screen-and-roll actions may facilitate shots near the rim or create additional space for ball handlers and perimeter shooters (18, 19). Increased defensive attention in the paint may also open weak-side catch-and-shoot opportunities through inside-out ball movement (20). By contrast, lineups paired with dominant shooting guards showed lower offensive TOV%. Concentrating ball-handling and late-clock decision-making in a primary creator may clarify possession responsibilities and allow local guards to operate more frequently off the ball, potentially reducing turnovers generated by distributed or less experienced creation (12, 21, 22).

Mid-to-long-range swingmen were associated with lower opponent eFG% and higher ORB%. Their combination of length, mobility, and perimeter orientation may support closeouts, switching, and pick-and-roll containment while preserving weak-side coverage (13, 23, 24); offensively, the same starting positions may facilitate weak-side crashes and access to longer rebound trajectories following perimeter attempts (25). Versatile bigs were associated with higher DRB% and FTr%. Their mobility may facilitate recovery to rebounding positions after perimeter defensive actions (18, 26–28), whereas screen-and-roll finishes, interior seals, mismatch attacks, and put-back attempts provide plausible pathways to greater foul pressure (5, 29).

Collectively, these patterns support a functional-fit interpretation of foreign-player integration: no single foreign-player profile was associated with favorable values across all efficiency indicators, and the relevance of a given role depended on the requirements of the local-player core. This perspective also suggests that foreign-player integration may redistribute local players' tactical responsibilities and expand the range of viable tactical configurations. A ball-dominant foreign guard, for example, may shift local guards toward off-ball functions, whereas an interior foreign player may alter spacing, screening, and rebounding roles; however, the present design cannot determine whether such short-term role reallocations facilitate or constrain long-term local-player development. From a policy perspective, the findings indicate that foreign-player regulations may shape the functional configurations available to teams, but competitive balance was not measured and no policy effect can be inferred. Moreover, the same foreign-player role may be deployed differently according to team system, coaching philosophy, pace of play, opponent characteristics, game state, and substitution context. The CART findings should therefore be viewed as exploratory lineup-matching patterns rather than intrinsic effects of specific foreign-player roles.

### 4.4 Limitations

Several limitations should be considered. First, excluding players below the prespecified playing-time threshold improved exposure stability but may have favored established rotation players. Second, the CART analyses were based on a limited number of aggregated lineup combinations and were explicitly exploratory; larger samples are required to assess the reproducibility of the observed role-efficiency patterns. Third, lineup structures were observed under the CBA foreign-player regulations in force during the study period, which may limit generalizability to leagues with different roster and substitution rules. Finally, opponent strength, game location, pace of play, game state, schedule effects, coaching philosophy, and team-specific tactical systems were not modeled explicitly. Because these factors may influence both lineup selection and efficiency, residual confounding cannot be excluded. Future research should incorporate larger multi-season samples, richer contextual information, and additional physiological or biomechanical indicators where appropriate to refine player profiles and test the stability of these lineup patterns across competitive environments.

## 5 Conclusion

This study redefined 10 local player roles and 7 foreign player roles using anthropometric, shooting-distance, and play-type data from three CBA seasons. Association rules showed that lineups featuring a ball handler and a paint anchor consistently included a 3D player, and always incorporated at least one on-ball scorer. Players with narrow technical profiles were not favored. The CART-derived patterns further indicated that lineup-efficiency indicators varied across foreign-player profiles. Lineups paired with a heavy big were characterized by higher offensive eFG%, those paired with a dominant shooting guard by lower offensive TOV%, those paired with a mid-to-long-range swingman by lower opponent eFG%, and those paired with a versatile big by higher DRB% and FTr%. These sample-specific associations suggest that foreign-player recruitment and deployment may be better considered in relation to the functional requirements of the existing local-player core. The findings extend role-based performance analysis from individual classification to lineup-level functional compatibility while remaining contingent on the tactical and regulatory context of the CBA.

## Data Availability

The raw data supporting the conclusions of this article will be made available by the authors, without undue reservation.

## References

[1] McBasketball C. Nylon Calculus: How to understand Synergy play type categories. Httpsfansided Com20170908nylon-Calc-Understandingsynergy-Play-Type-Data (2017). Available online at: https://fansided.com/2017/09/08/nylon-calculus-understanding-synergy-play-type-data/ (Accessed February 27, 2024)

[2] Božović B. The use of “synergy sports technology” for the collection of basketball game statistics. In: Sinteza 2021-international scientific conference on information technology and data related research, Singidunum University (2021), 272–6. 10.15308/Sinteza-2021-272-276

[3] Chen R Zhang M Xu X. Modeling the influence of basketball players' Offense roles on team performance. Front Psychol. (2023) 14:1256796. 10.3389/fpsyg.2023.1256796 37744596 PMC10514490

[4] hang S Lorenzo A Gómez M-A Mateus N Gonçalves B Sampaio J. Clustering performances in the NBA according to players' Anthropometric attributes and playing experience. J Sports Sci. (2018) 36:2511–20. 10.1080/02640414.2018.1466493 29676222

[5] Wang X Han B Zhang S Zhang L Calvo AL Gomez M-Á. The differences in the performance profiles between native and foreign players in the Chinese basketball association. Front Psychol. (2022) 12:788498. 10.3389/fpsyg.2021.788498 35173649 PMC8842947

[6] Shea SM Baker CE. Basketball analytics: objective and efficient strategies for understanding how teams win. Advanced Metrics. (2013).

[7] Wei B Li Z Zhou C Gómez M-Á Zhang M. Enhancing team dynamics: unveiling synergy effects through social network analysis in professional basketball. Acta Psychol (Amst). (2025) 261:105747. 10.1016/j.actpsy.2025.105747 41109113

[8] Christos K Dimitrios L Christos G. Effect of offensive rebound on the game outcome during the 2019 Basketball World Cup. J Phys Edu Sport (2020) 20:3651. 10.7752/jpes.2020.06492

[9] Shawkat M Badawi M El-ghamrawy S Arnous R El-desoky A. An optimized FP-growth algorithm for discovery of association rules. J Supercomput. (2022) 78:5479–506. 10.1007/s11227-021-04066-y

[10] Kavakiotis I Tsave O Salifoglou A Maglaveras N Vlahavas I Chouvarda I. Machine learning and data mining methods in diabetes research. Comput Struct Biotechnol J. (2017) 15:104–16. 10.1016/j.csbj.2016.12.005 28138367 PMC5257026

[11] Sampaio J McGarry T Calleja-González J Jiménez Sáiz S Schelling I Del Alcázar X Balciunas M. Exploring game performance in the National Basketball Association using player tracking data. PLoS One. (2015) 10:e0132894. 10.1371/journal.pone.0132894 26171606 PMC4501835

[12] Ozmen MU. Foreign player quota, experience and efficiency of basketball players. J Quant Anal Sports. (2012) 8:1–18. 10.1515/1559-0410.1370

[13] Teramoto M Cross CL. Importance of team height to winning games in the national basketball association. Int J Sports Sci Coach. (2018) 13:559–68. 10.1177/1747954117730953

[14] Zukolo Z Dizdar D Selmanović A Vidranski T. The role of finishing actions in the final result of the basketball match. J Sports Sci. (2019) 12:90–5.

[15] Matulaitis K Bietkis T. Prediction of offensive possession ends in elite basketball teams. Int J Environ Res Public Health. (2021) 18:1083. 10.3390/ijerph18031083 33530475 PMC7908613

[16] Kubatko J Oliver D Pelton K Rosenbaum DT. A starting point for analyzing basketball statistics. J Quant Anal Sports. (2007) 3:1–22. 10.2202/1559-0410.1070

[17] Rousseeuw PJ. Silhouettes: a graphical aid to the interpretation and validation of cluster analysis. J Comput Appl Math. (1987) 20:53–65. 10.1016/0377-0427(87)90125-7

[18] Çene E. What is the difference between a winning and a losing team: insights from Euroleague basketball. Int J Perform Anal Sport. (2018) 18:55–68. 10.1080/24748668.2018.1446234

[19] Lorenzo J Lorenzo A Conte D Giménez M. Long-term analysis of elite basketball players' game-related statistics throughout their careers. Front Psychol. (2019) 10:421 10.3389/fpsyg.2019.00421 30873096 PMC6400965

[20] Koon Teck K Wang C Mallett C. Discriminating factors between successful and unsuccessful elite youth Olympic female basketball teams. Int J Perform Anal Sport. (2012) 12:119–31. 10.1080/24748668.2012.11868588

[21] Gasperi L Conte D Leicht A Gómez-Ruano M-Á. Game related statistics discriminate national and foreign players according to playing position and team ability in the women's basketball EuroLeague. Int J Environ Res Public Health. (2020) 17:5507. 10.3390/ijerph17155507 32751559 PMC7432202

[22] Paulauskas R Kasparavicius R Stumbras M Figueira B. Comparative analysis of national and foreign players' performance in euroleague basketball. PLoS One. (2024) 19:e0306240. 10.1371/journal.pone.0306240 39163411 PMC11335165

[23] Rangel W Ugrinowitsch C Lamas L. Basketball players' versatility: assessing the diversity of tactical roles. Int J Sports Sci Coach. (2019) 14:552–61. 10.1177/1747954119859683

[24] Nikolaidis PT Asadi A Santos EJ Calleja-González J Padulo J Chtourou H. Relationship of body mass status with running and jumping performances in young basketball players. Muscles Ligaments Tendons J. (2015) 5:187. 10.32098/mltj.03.2015.08 26605193 PMC4617219

[25] Ou-Yang Y Hong W Peng L Mao C-X Zhou W-J Zheng W-T. Explaining basketball game performance with SHAP: insights from Chinese basketball association. Sci Rep. (2025) 15:13793. 10.1038/s41598-025-97817-3 40258968 PMC12012039

[26] Christmann J Akamphuber M Müllenbach AL Güllich A. Crunch time in the NBA—the effectiveness of different play types in the endgame of close matches in professional basketball. Int J Sports Sci Coach. (2018) 13:1090–9. 10.1177/1747954118772485

[27] Sampaio J Janeira M Ibáñez S Lorenzo A. Discriminant analysis of game-related statistics between basketball guards, forwards and centres in three professional leagues. Eur J Sport Sci. (2006) 6:173–8. 10.1080/17461390600676200

[28] Zarić I Kukić F Jovićević N Zarić M Marković M Toskić L. Body height of elite basketball players: do taller basketball teams rank better at the FIBA World Cup? Int J Environ Res Public Health. (2020) 17:3141. 10.3390/ijerph17093141 PMC7246476 32365985

[29] Oliver D. Basketball on Paper: Rules and Tools for Performance Analysis. Potomac Books Inc.: U of Nebraska Press (2004).

